# Addition of docetaxel to S-1 results in significantly superior 5-year survival outcomes in Stage III gastric cancer: a final report of the JACCRO GC-07 study

**DOI:** 10.1007/s10120-023-01419-9

**Published:** 2023-08-07

**Authors:** Yasuhiro Kodera, Kazuhiro Yoshida, Mitsugu Kochi, Takeshi Sano, Wataru Ichikawa, Yoshihiro Kakeji, Yu Sunakawa, Masahiro Takeuchi, Masashi Fujii

**Affiliations:** 1 Department of Gastroenterological Surgery, Nagoya University Graduate School of Medicine, 65, Tsurumai-cho, Showa-ku, Nagoya, 466-8550 Japan; 2 Department of Surgical Oncology, Gifu University, Gifu, Japan; 3 Department of Hepato-Biliary-Pancreatic and Gastrointestinal Surgery, School of Medicine, International University of Health and Welfare, Ichikawa, Japan; 4 Gastroenterological Surgery, Cancer Institute Hospital, Tokyo, Japan; 5 Division of Medical Oncology, Showa University Fujigaoka Hospital, Yokohama, Japan; 6 Division of Gastrointestinal Surgery, Department of Surgery, Kobe University Graduate School of Medicine, Kobe, Japan; 7 Department of Clinical Oncology, St. Marianna University School of Medicine, Kawasaki, Japan; 8 The University of Tokyo Graduate School of Mathematical Sciences, Tokyo, Japan; 9 Department of Digestive Surgery, Nihon University School of Medicine, Tokyo, Japan

**Keywords:** S-1, Docetaxel, Gastric cancer, Adjuvant chemotherapy, Stage III

## Abstract

**Purpose:**

A phase III trial comparing S-1 and docetaxel with S-1 alone as postoperative chemotherapy for pathologically Stage III gastric cancer was conducted and clarified the superiority of the doublet in terms of 3-year relapse-free survival as the primary endpoint (67.7% versus 57.4%, hazard ratio [HR] 0.715, 95% confidence interval [CI] 0.587–0.871; *p* = 0.0008). This final report analyzed 5-year survival outcomes along with the incidence and pattern of late recurrences.

**Patients and methods:**

Patients with histologically confirmed Stage III gastric cancer who underwent gastrectomy with D2 lymphadenectomy were randomly assigned to receive adjuvant chemotherapy with either S-1 plus docetaxel or S-1 alone. The same 912 patients who were evaluated for 3-year survival outcomes in the previous report were analyzed.

**Results:**

Five-year overall survival rate of the S-1 plus docetaxel group (67.91%) was significantly superior to that in the S-1 group (60.27%; HR 0.752, 95% CI 0.613–0.922; *p* = 0.0059). The incidence of late recurrence at > 3 years after randomization was similar in both groups (7.3% versus 7.2%). Peritoneal dissemination was the most common pattern of late recurrence. Addition of docetaxel significantly suppressed relapse through the lymphatic (6.8% [95% CI 4.52–9.17] versus 15% [95% CI 11.76–18.30]; *p* < 0.0001) and hematogenous (10.2% [95% CI 7.37–12.94] versus 15.7% [95% CI 12.36–19.01]; *p* < 0.0137) pathways throughout the 5 years of follow-up.

**Conclusion:**

The survival benefit of postoperative chemotherapy with S-1 and docetaxel in terms of 5-year overall survival rate was confirmed for patients with pathologically Stage III gastric cancer, although late recurrences were not prevented.

## Introduction

The prognosis of patients with gastric cancer is dismal once recurrence occurs after curative surgery. Optimal adjuvant treatments to avoid recurrence have been explored extensively, resulting in some differences in current standards of care between the West and Far East [1]. Due to the relatively favorable outcomes for patients owing to screening and widespread use of D2 dissection, treatment with upfront surgery followed by postoperative adjuvant chemotherapy remains the standard treatment for patients with clinically Stage II/III gastric cancer in Japan [2].

JACCRO GC-07 is a randomized phase III study examining the superiority of S-1 plus docetaxel over S-1 monotherapy in the postoperative adjuvant setting for patients with pathologically Stage III gastric cancer who underwent D2 gastrectomy. Since the primary endpoint of 3-year relapse-free survival was met both at the second interim analysis [3] and at the time all patients had completed 3 years of follow-up [4], the doublet has been regarded in Japanese guidelines as a standard treatment for this population [5].

In this short communication, we report on overall survival (OS) and relapse-free survival (RFS) rates at 5 years after surgery, as the last two secondary endpoints in this trial. In addition, we sought to explore whether the addition of docetaxel had any preventive effects on late recurrences diagnosed > 3 years after randomization.

## Patients and methods

### Patients and interventions

Details of this randomized phase III trial have already been published [3, 4]. In brief, patients were deemed eligible when they were aged 20–80 years, underwent R0 resection by ≥ D2 gastrectomy, and were confirmed from operative findings and histopathological examination of resected specimens to have Stage III gastric cancer as defined by the third English edition of the Japanese Classification of Gastric Carcinoma [6], in which the staging is identical to that for stomach cancer in the seventh edition of the TNM classification [7]. Patients in the control group were randomized to receive 12 months of S-1 monotherapy (at a dose of 120 mg/day for 4 weeks with 2 weeks of rest for patients with body surface area ≥ 1.5 m^2^, 100 mg for patients with body surface area of 1.25–1.5 m^2^, and 80 mg for patients with body surface area ≤ 1.25 m^2^). Patients in the test arm were to be treated with a triweekly combination regimen comprising oral S-1 (2 weeks at the same dosage as controls, with 1 week of rest) with intravenous docetaxel at 40 mg/m^2^ on day 1. Six cycles of this combination were to be delivered after one cycle of S-1 monotherapy (2-week administration followed by 1 week of rest), to be followed by further S-1 monotherapy for a total of 12 months [2]. The protocol treatment was to be started within 42 days postoperatively.

Patients were to be followed-up for 5 years. The method of follow-up has been described previously [3] and was in accordance with the recommendations provided in the Japanese Gastric Cancer Treatment Guidelines [5].

### Outcomes

The primary endpoint was 3-year RFS. Secondary endpoints were 3-year OS rate, 5-year OS rate, 5-year RFS rate, and incidence of adverse events. OS is defined as the time from randomization to death from all causes. RFS is defined as the time from randomization to either disease recurrence or death from all causes. Recurrence was judged by imaging diagnosis and/or clinical relapse on the basis of non-imaging exacerbation of disease conditions. Late recurrence at > 3 years after surgery was analyzed independently and incidences were compared between the two arms.

### Study design and statistical analysis

Sample size calculations were based on data from the previous phase III study^2^, in which the 3-year RFS rate was 62% for Stage III disease when treated using postoperative S-1 monotherapy. The sample size had originally been set at 1100 patients (550 per group), with an expectation that the 3-year RFS rate would improve by 7% with the addition of docetaxel. However, that study was terminated prematurely after detecting a more substantial survival benefit of the combination therapy at the interim analysis with only 915 patients^3^. As described in the previous report, further 3 patients were excluded due to ineligibility^4^, with the remaining 912 patients being entered into the present analyses.

Cumulative survival curves and annual survival rates were estimated using Kaplan–Meier curves. Between-group analyses were conducted using stratified log-rank tests. Hazard ratios (HRs) and two-sided 95% confidence intervals (CIs) were estimated using the Cox proportional hazards regression modeling.

## Results

Final survival analyses were conducted at a median follow-up of 63.7 months (range: 3.5–111.9 months). Time from the date of randomization of the last enrolled case to the data cutoff date was 66.8 months. Data from the 55 patients lost to follow-up within 5 years from the date of random assignment were censored as of the last day of follow-up.

**Fig. 1:**
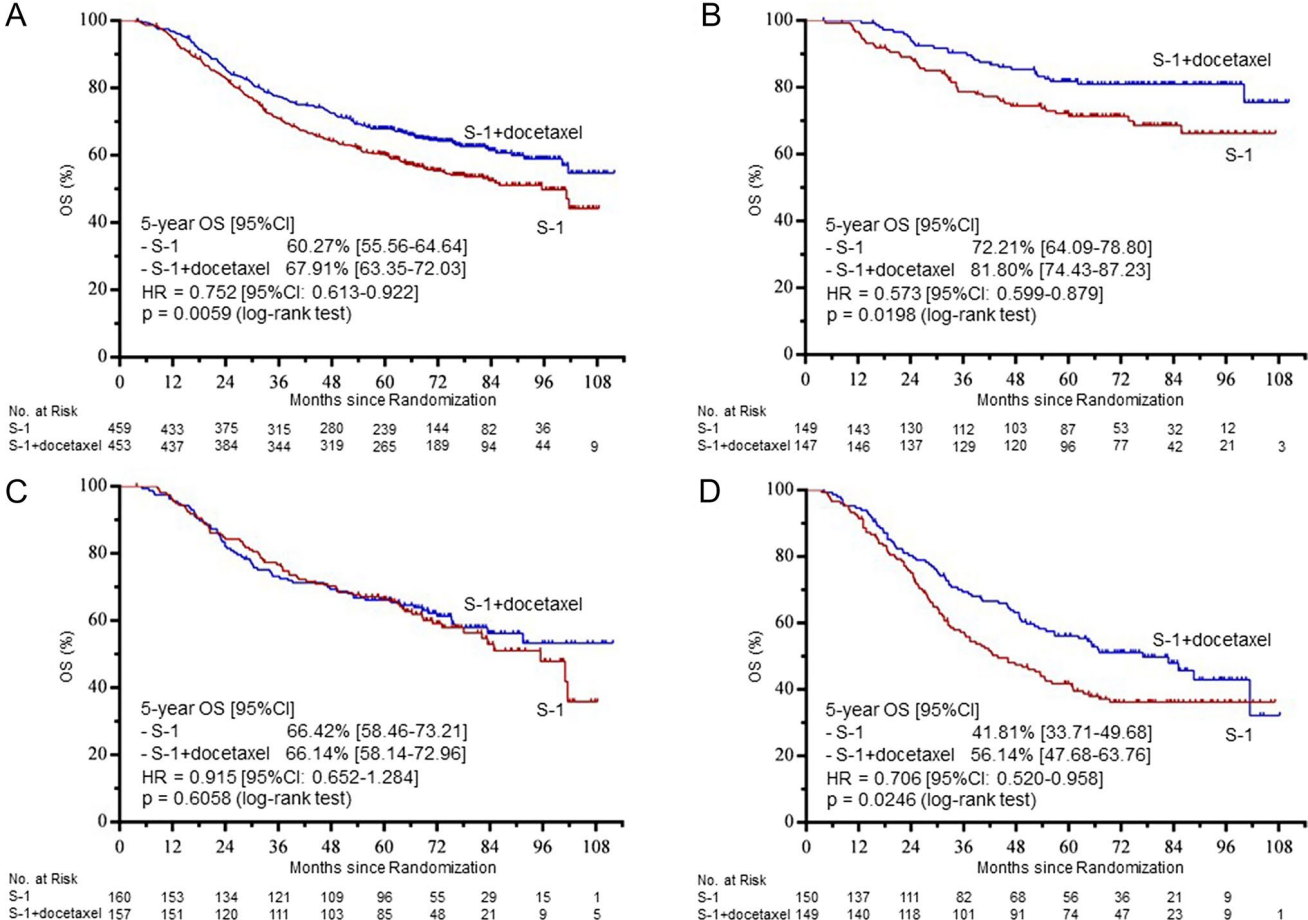
Overall survival (OS) at termination of the preplanned follow-up for 5 years. Kaplan–Meier estimates of OS in all patients (**A**) and in patients with Stage IIIA (**B**), IIIB (**C**), and IIIC disease (**D**)

Among the 912 patients, 190 patients in the S-1 plus docetaxel group and 236 patients in the S-1 monotherapy group showed recurrence during the 5 years of follow-up, with the death of 167 patients and 206 patients, respectively. Five-year OS rates were 67.91% in the S-1 plus docetaxel group and 60.27% in the S-1 monotherapy group (HR 0.752, 95% CI 0.613–0.922; *p* = 0.0059). Five-year OS rates for patients with Stage IIIA disease were 81.80% and 72.21%, respectively (HR 0.573, 95%CI 0.357–0.921; *p* = 0.0198). Five-year OS rates for patients with Stage IIIC disease were 56.14% and 41.81%, respectively (HR 0.706, 95%CI 0.520–0.958; *p* = 0.0246). However, no significant improvement in survival was observed for patients with Stage IIIB disease, with 5-year OS rates of 66.14% and 66.42%, respectively (HR 0.915, 95% CI 0.652–1.284; *p* = 0.6058) (Fig. 1).

**Fig. 2:**
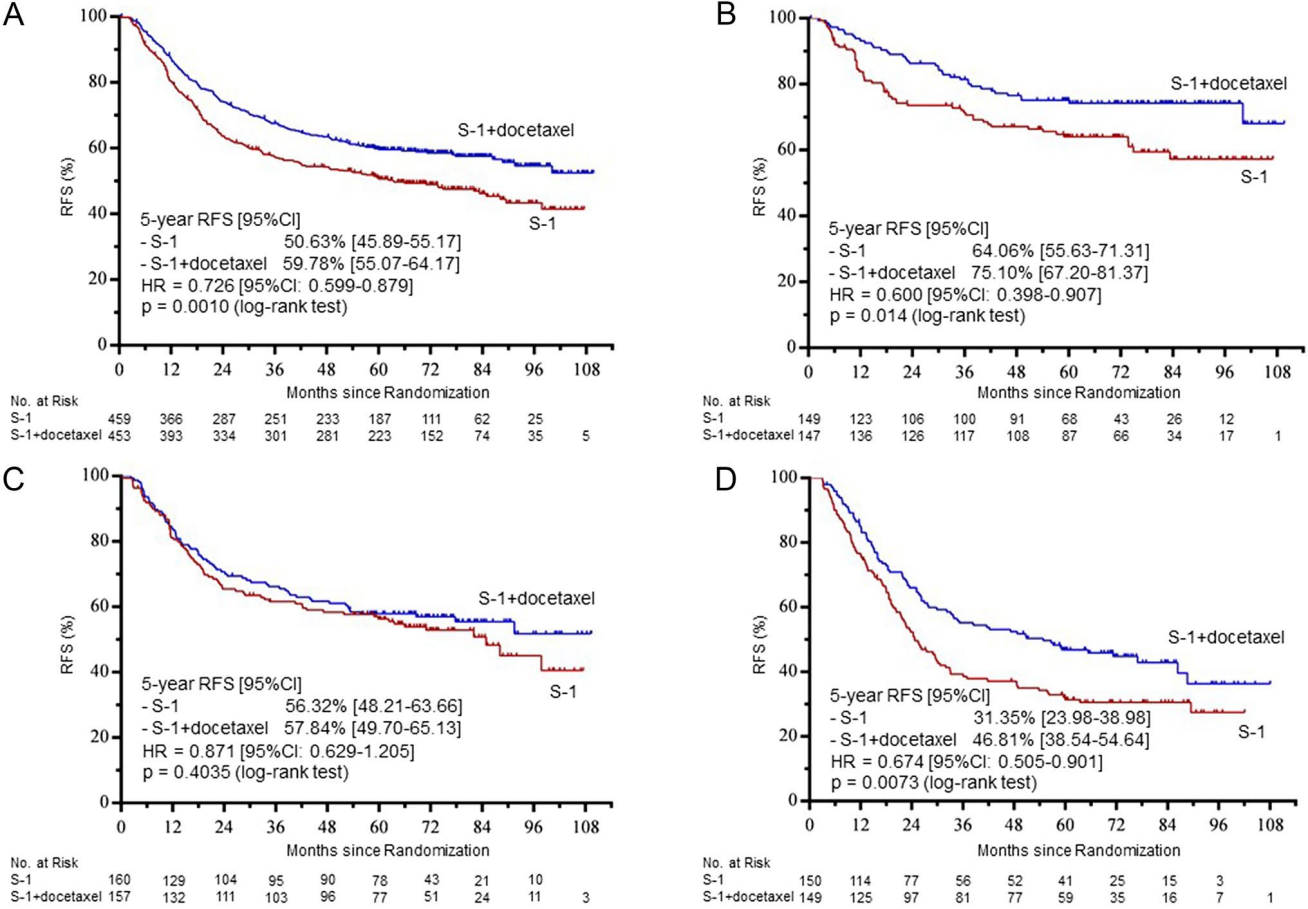
Relapse-free survival (RFS) at termination of the preplanned follow-up for 5 years. Kaplan–Meier estimates of RFS in all patients (**A**) and in those with Stage IIIA (**B**), IIIB (**C**), and IIIC disease (**D**)

Five-year RFS rates were 59.78% in the S-1 plus docetaxel group and 50.63% in the S-1 group (HR 0.726, 95%CI 0.599–0.879; *p* = 0.0010). Five-year RFS rates in patients with Stage IIIA, IIIB, and IIIC gastric cancer were 75.10% and 64.06% (HR 0.600, 95% CI 0.308–0.907; *p* = 0.0140), 57.84% and 56.32% (HR 0.871, 95% CI 0.629–1.205; *p* = 0.4035), and 46.81% and 31.35% (HR 0.674, 95% CI 0.505–0.901; *p* = 0.0073), respectively (Fig. 2).

The number of patients with late recurrence diagnosed > 3 years after randomization was similar in each treatment arm (Table 1). The most common site of late recurrence in both groups was the peritoneal surface. Recurrences through lymphatic and hematogenous routes, along with the total number of recurrences, remained significantly suppressed by the addition of docetaxel during the 5 years of follow-up (Table 2). There were no differences, either in the number of deaths from other cancer or disease during the 5 years of follow-up (5 and 16 for the doublet group and 6 and 14 for the S-1 monotherapy group, respectively).

**Table 1:** Sites of relapse at ≤ 3 years and > 3 years after randomization

	≤ 3 years from randomization		> 3 years from randomization	
	S-1 (*n* = 459)	S-1/docetaxel (*n* = 453)	S-1 (*n* = 459)	S-1/docetaxel (*n* = 453)
No. of pts. with relapse	188	136	33	33
Local	16	11	4	2
Lymph nodes	63	24	6	7
Peritoneum	85	75	19	14
Hematogenous	64	37	8	9
Others	6	4	2	2

Some patients recorded more than one metastatic site

**Table 2:** Site of relapse during the 5 years after randomization, stratified by treatment arm

	S-1 (*n* = 459)		S-1/docetaxel (*n* = 453)		*P*
	*n* (%)	95%CI	*n* (%)	95%CI	
No. of pts. with relapse	221 (48.1%)	43.58–52.72	169 (37.3%)	32.85–41.76	0.0009
Local	20 (4.4%)	2.49–6.22	13 (2.9%)	1.33–4.41	0.2876
Lymph nodes	69 (15.0%)	11.76–18.30	31 (6.8%)	4.52–9.17	< 0.0001
Peritoneum	104 (22.7%)	18.83–26.49	89 (19.6%)	15.99–23.31	0.2920
Hematogenous	72 (15.7%)	12.36–19.01	46 (10.2%)	7.37–12.94	0.0137

Incidence of relapse tended to be lower in the S-1 plus docetaxel arm for all patterns of metastasis, with significant differences in the incidence of relapse through lymphatic and hematogenous routes

Details on adverse events and compliance with each of the two drug regimens have been reported in our previous studies [3, 4]. No further data in this category were accumulated.

Regarding the histological type, recurrences were more frequent among the undifferentiated type at 261 of 544 patients (48%) as opposed to the differentiated type (125 of 368 patients, 34%). Nevertheless, similar risk reduction for relapse was observed across the two types through addition of docetaxel (HR 0.743, 95% CI 0.531–1.038; *p* = 0.0805 for the differentiated type and HR 0.714, 95% CI 0.565–0.901; *p* = 0.0043 for the undifferentiated type).

## Discussion

Through several attempts to optimize the triplet of fluorouracil, cisplatin, and docetaxel after the V325 study [8], and more recently with emergence of the FLOT regimen [9], docetaxel has become a key drug in the treatment of gastric cancer alongside fluorouracil and platinum agents. Differences in toxicity profiles between docetaxel and platinum agents and the limited tolerance of Japanese post-gastrectomy patients for intensive chemotherapeutic regimens prompted us to explore the combination of S-1 and docetaxel in the postoperative adjuvant setting.

The present study confirmed the superior efficacy of S-1 plus docetaxel over S-1 monotherapy for patients with pathologically Stage III gastric cancer based on the 5-year survival outcomes of a phase III study. This trend was observed in both the differentiated and undifferentiated type. Although reasons for the lack of differences in long-term survival among the Stage IIIB population^4^ remain enigmatic, the combination in question has shown significant survival benefits for both Stage IIIA and Stage IIIC subsets. Analysis at 5 years from randomization confirmed that the addition of docetaxel led to significant decreases in hematogenous recurrences alongside reinforcement or preservation of efficacy against both lymphatic spread and peritoneal dissemination; effects already observed in treatment with S-1 alone [2]. However, these benefits were observed exclusively during the first 3 years and the present analyses revealed no benefit of the doublet in preventing late recurrences at > 3 years from randomization.

Other doublets consisting of fluorouracil and platinum agents are available for postoperative adjuvant chemotherapy. More recently, 6 months of S-1 and oxaliplatin was found to be superior to 12 months of S-1 monotherapy in a Korean phase III trial [10], and was found to be non-inferior to 6 months of capecitabine and oxaliplatin in a Chinese phase III trial [11]. In the absence of head-to-head comparisons between docetaxel-containing and oxaliplatin-containing doublets, the most appropriate regimen should be selected carefully on a case-by-case basis based on the toxicity profile and intended duration of treatment. Among other adverse events, docetaxel is typically associated with alopecia, whereas patients treated with oxaliplatin often suffer from neurotoxicity.

In conclusion, the evidence recommending postoperative chemotherapy with S-1 and docetaxel is robust for patients with pathologically Stage III gastric cancer who undergo D2 resection, regardless of the histological type, although late recurrences at > 3 years after randomization remain unpreventable.

## Data Availability

The data that support the findings of this study are not openly available due to reasons of sensitivity and are available from Japan Clinical Cancer Research Organization (jaccro@jaccro.or.jp) upon reasonable request. Data are located in controlled access data storage at Japan Clinical Cancer Research Organization.
