# Correction: Addition of docetaxel to S-1 results in significantly superior 5-year survival outcomes in Stage III gastric cancer: a final report of the JACCRO GC-07 study

**DOI:** 10.1007/s10120-024-01519-0

**Published:** 2024-06-12

**Authors:** Yasuhiro Kodera, Kazuhiro Yoshida, Mitsugu Kochi, Takeshi Sano, Wataru Ichikawa, Yoshihiro Kakeji, Yu Sunakawa, Masahiro Takeuchi, Masashi Fujii

**Affiliations:** 1 Department of Gastroenterological Surgery, Nagoya University Graduate School of Medicine, 65, Tsurumai-cho, Showa-ku, Nagoya, 466-8550 Japan; 2 Department of Surgical Oncology, Gifu University, Gifu, Japan; 3 Department of Hepato-Biliary-Pancreatic and Gastrointestinal Surgery, School of Medicine, International University of Health and Welfare, Ichikawa, Japan; 4 Gastroenterological Surgery, Cancer Institute Hospital, Tokyo, Japan; 5 Division of Medical Oncology, Showa University Fujigaoka Hospital, Yokohama, Japan; 6 Division of Gastrointestinal Surgery, Department of Surgery, Kobe University Graduate School of Medicine, Kobe, Japan; 7 Department of Clinical Oncology, St. Marianna University School of Medicine, Kawasaki, Japan; 8 The University of Tokyo Graduate School of Mathematical Sciences, Tokyo, Japan; 9 Department of Digestive Surgery, Nihon University School of Medicine, Tokyo, Japan

**Correction: Gastric Cancer (2023) 26:1063–1068** 10.1007/s10120-023-01419-9

The Nov, 2023 article by Kodera et al. entitled “Addition of docetaxel to S-1 results in significantly superior 5-year survival outcomes in Stage III gastric cancer: a final report of the JACCRO GC-07 study” (Gastric Cancer 10.1007/s10120-023-01419-9) was published online on August 7, 2023, with errors.

The first sentence of the second paragraph in the Results section read as: Among the 912 patients, 190 patients in the S-1 plus docetaxel group and 236 patients in the S-1 monotherapy group showed recurrence during the 5 years of follow up, with the death of 167 patients and 206 patients, respectively.

It should have read as: During the 5 years of follow up, 426 events had been observed; 190 events in the S-1 plus docetaxel group and 236 events in the S-1 monotherapy group.

This has been corrected as of May 22, 2024. The authors apologize for the errors.

